# ADATs: roles in tRNA editing and relevance to disease: tRNA editing by ADATs

**DOI:** 10.3724/abbs.2024125

**Published:** 2024-07-22

**Authors:** Xue-Ling Mao, Gilbert Eriani, Xiao-Long Zhou

**Affiliations:** 1 Key Laboratory of RNA Innovation Science and Engineering CAS Center for Excellence in Molecular Cell Science Shanghai Institute of Biochemistry and Cell Biology Chinese Academy of Sciences University of Chinese Academy of Sciences Shanghai 200031 China; 2 Architecture et Réactivité de l′ARN Institut de Biologie Moléculaire et Cellulaire du CNRS Université de Strasbourg 2 allée Konrad Roentgen 67084 Strasbourg France; 3 Key Laboratory of Systems Health Science of Zhejiang Province School of Life Science Hangzhou Institute for Advanced Study University of Chinese Academy of Sciences Hangzhou 310024 China

**Keywords:** tRNA editing, deaminase, TadA, ADAT2/3

## Abstract

Transfer RNAs (tRNAs) play central roles in protein biosynthesis. Post-transcriptional RNA modifications affect tRNA function and stability. Among these modifications, RNA editing is a widespread RNA modification in three domains of life. Proteins of the adenosine deaminase acting on tRNA (ADAT) family were discovered more than 20 years ago. They catalyze the deamination of adenosine to inosine (A-to-I) or cytidine to uridine (C-to-U) during tRNA maturation. The most studied example is the TadA- or ADAT2/3-mediated A-to-I conversion of the tRNA wobble position in the anticodon of prokaryotic or eukaryotic tRNAs, respectively. This review provides detailed information on A-to-I and C-to-U editing of tRNAs in different domains of life, presents recent new findings on ADATs for DNA editing, and finally comments on the association of mutations in the *ADAT3* gene with intellectual disability.

## Introduction

**Figure 1:**
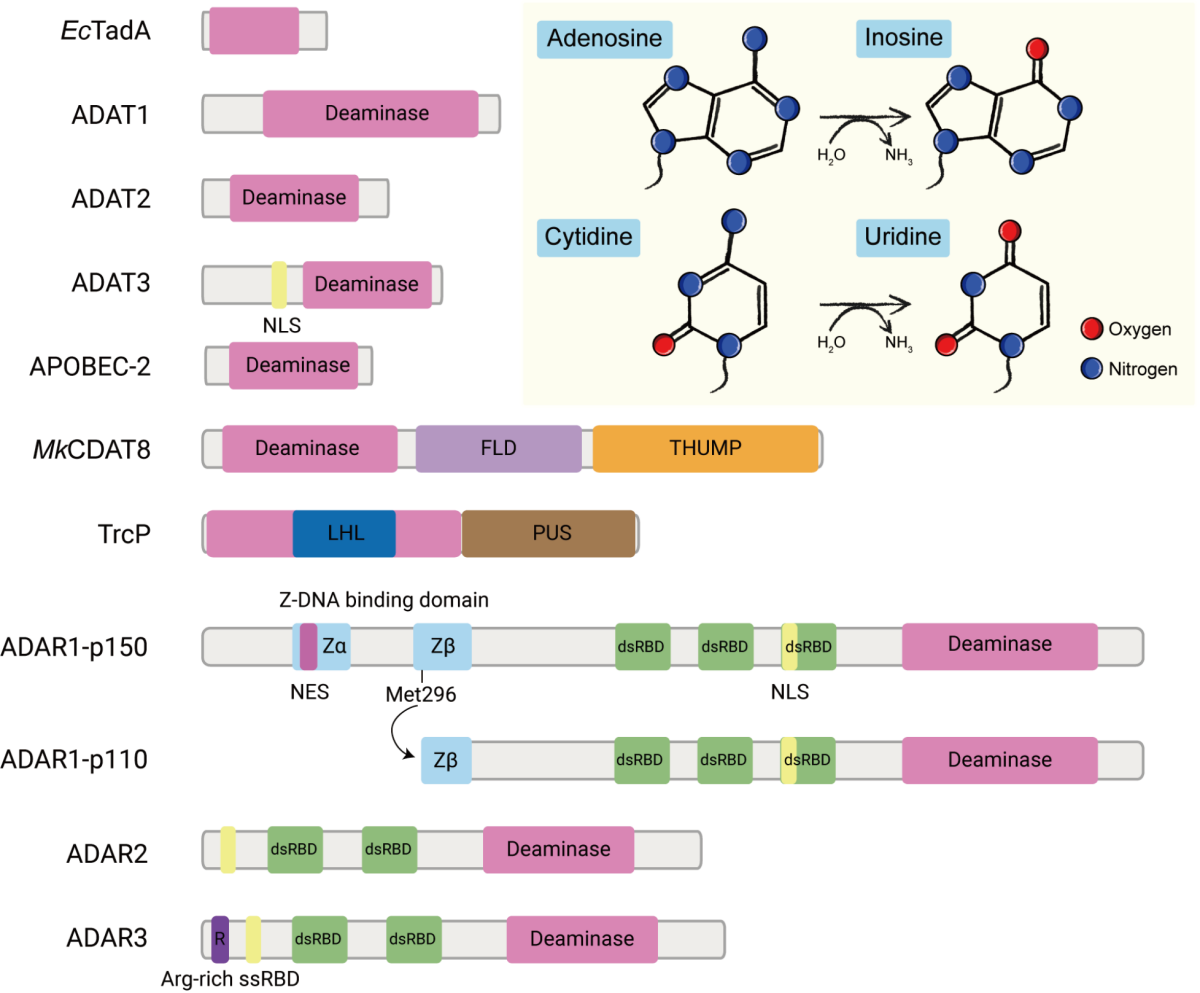
Figure 1 Domain structures of the RNA deaminase family and the A-to-I and C-to-U deamination reactions The deaminase domain is in pink; the nuclear location signal (NLS) is in yellow; the nuclear export signal (NES) is in red-purple; the ferredoxin-like domain (FLD) is in purple; the THUMP domain is in orange; the long helical (LHL) domain is in dark blue; the pseudouridine synthase (PUS) domain is in brown; the Z-DNA binding domain is in blue; the double-stranded RNA binding domain (dsRBD) is in green; and the arginine-rich single-stranded RNA binding domain (Arg-rich ssRBD) is in dark purple.

RNA is a critical biomolecule in genetic information decoding and undergoes a series of metabolic processes after transcription, such as modification, maturation, and degradation, for normal life activities. Modified RNA nucleotides other than the four standard nucleotides have fascinated scientists since the identification of pseudouridine (ψ) as the “fifth” ribonucleotide in 1951 [1]. To date, more than 170 different types of modified ribonucleosides have been identified in RNAs from all three domains of life [ 2, 3], and the majority of modifications occur on transfer RNA (tRNA) molecules [ 4, 5]. tRNAs are short, ubiquitous adaptor molecules that play a central role in connecting genetic information with the protein synthesizing machinery. RNA editing is a unique modification reaction that changes RNA fate and the genomic sequence. The most common type of RNA editing is the deamination of adenosine to inosine (A-to-I) or cytidine to uridine (C-to-U) in mRNAs and non-coding RNAs ( Figure 1), which expands the genetic diversity beyond the DNA-encoded sequence or has other biological significance.

**Figure 2:**
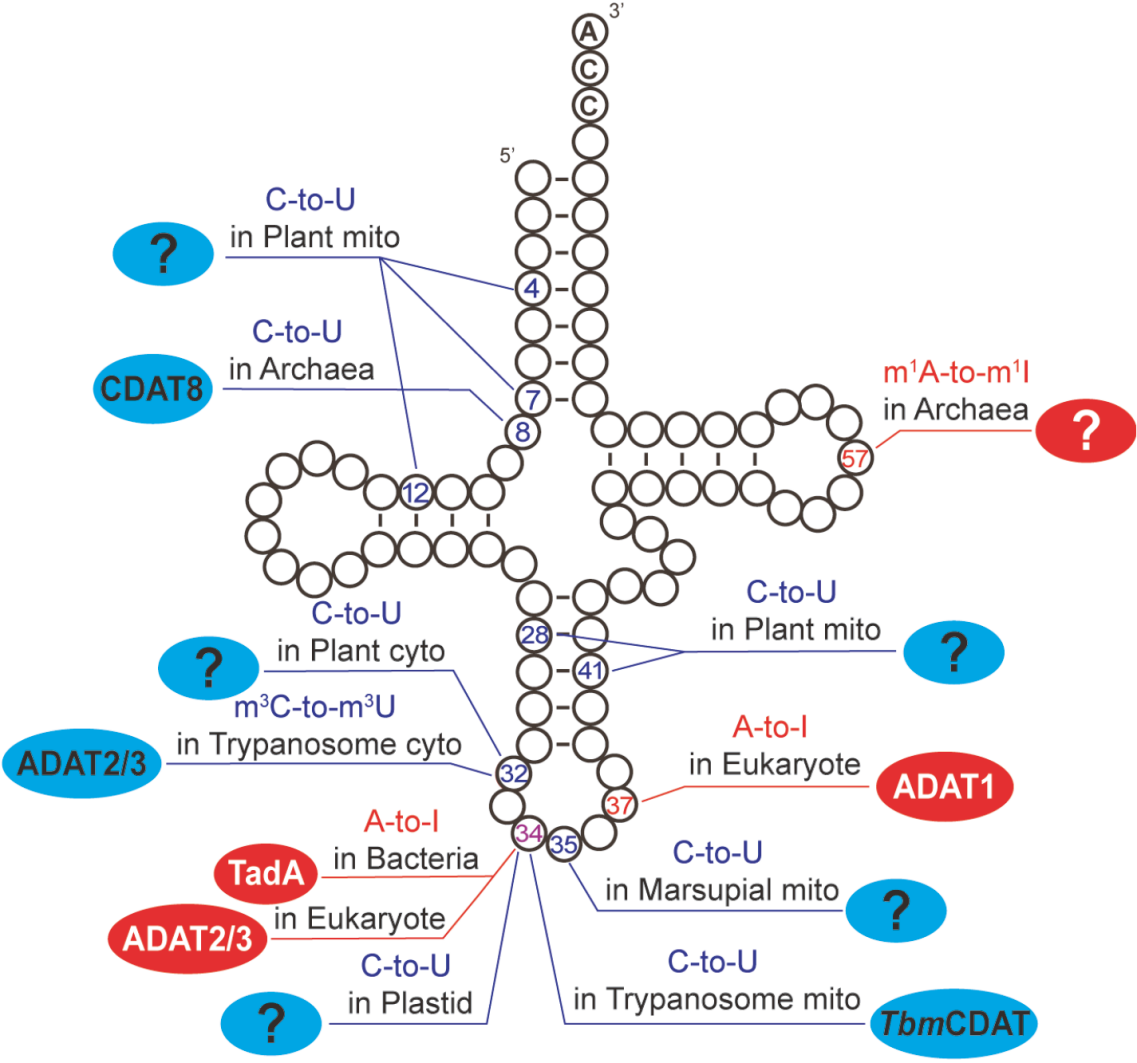
Figure 2 Schematic overview of tRNA editing across three branches of life The locations of A-to-I editing and C-to-U editing are shown in red and blue, respectively. The colored oval circles indicate the genes involved in the changes (when identified). Mito, mitochondria; cyto, cytoplasm.

Inosine was discovered in tRNA ^Ala^ as early as 1965 [6], but it was not until 1987 that an A-to-I RNA editing activity was observed as a double-stranded RNA (dsRNA) unwinding activity in *Xenopus laevis* oocytes and embryos [7]. The enzyme was subsequently identified as an RNA adenosine deaminase (ADAR) [8]. In addition to ADARs, adenosine deaminases, known as adenosine deaminases, which act on tRNAs (ADATs), function on tRNAs. In addition to carrying out A-to-I editing of tRNAs, which includes 1-methylinosine (m ^1^I) modifications in certain organisms, ADATs also catalyze C-to-U deamination ( Figure 2). Both ADARs and ADATs belong to the cytidine deaminase superfamily and require zinc ions for their activities. Although ADARs are well documented due to their crucial role in mRNA and have been the focus of several related studies [ 9‒ 12], ADATs are comparatively less understood.

In this review, we summarize the structural features, functions and evolutionary history of the ADATs within the RNA-dependent deaminase superfamily. We focus on the editing of tRNAs by ADATs, explaining A-to-I and C-to-U deamination processes across different organisms while highlighting their substrates and functions ( Figure 2). This review concludes with recent interesting findings that reveal the dual role of ADAT2/3 in RNA and DNA editing. It also covers the molecular mechanisms of ADAT3 mutations that cause intellectual disability, revealing a rare link between the ADAT family and disease.

## ADATs in the Superfamily of RNA-dependent Deaminases

ADATs comprise a group of enzymes responsible for catalyzing the deamination of tRNA molecules, including ADAT1, ADAT2 and ADAT3, which work in concert to achieve A-to-I or C-to-U editing in tRNAs. ADAT1, also known as Tad1 in yeast, is a homodimer and plays a central role in A-to-I deamination [13]. ADAT2 and ADAT3 form a heterodimer that catalyzes deamination. ADAT2, also known as Tad2, is considered the catalytic subunit, while ADAT3 (known as Tad3) serves as an essential co-factor in tRNA substrate recognition in eukaryotes [ 14, 15]. In bacteria, TadA forms a homodimer and performs the same enzymatic functions as ADAT2/3 [ 13, 16].

Structurally, ADATs are composed mainly of a deamination domain, except ADAT3 which also harbors a nuclear localization signal peptide at its N-terminus ( Figure 1) [14]. ADAT3, a cofactor of ADAT2, is also thought to contain a tRNA-binding structural domain [15] .

In comparison, the mammalian genome encodes three ADARs: ADAR1, ADAR2, and ADAR3. ADAR1 and ADAR2 are thought to have catalytic activity, while ADAR3 has no editing activity but has been found to inhibit the activity of ADAR1 and ADAR2 by competitively binding to RNA ( Figure 1) [10]. Each protein has a catalytic deaminase domain at the C-terminus followed by several double-stranded RNA binding domains (dsRBDs); ADAR2 and ADAR3 contain two dsRBDs, while ADAR1 has three dsRBDs. In ADAR1, the third dsRNA binding domain contains a nuclear localization sequence, whereas in ADAR2 and ADAR3, this sequence is located at the N-terminus. Furthermore, the N-terminus of ADAR3 accommodates an Arg-rich domain for binding with single-stranded RNA [17]. Two different types of ADAR1 are present—ADAR1 p110 and ADAR1 p150—which arise from separate gene promoter transcription [9]. At its N-terminus, ADAR1 p110 possesses a Z-DNA-binding domain referred to as Zβ. ADAR1 p150 harbors Zβ and an additional Zα domain that carries a nuclear export signal (NES), so it is located mainly in the cytoplasm and shuttles between the nucleus and the cytoplasm [18]. A recent study showed that Zα-domain proteins ensure coordination and interaction within the immune system [19]. The sequences of the ADATs and ADARs deaminase domains are highly homologous. ADARs are not present in prokaryotic, fungal, or plant genomes [20]. Instead, these genomes encode ADATs which are conserved in eukaryotes from yeast to humans. The presence of ADAT family proteins in prokaryotes, fungi, plants and higher eukaryotes indicates the functional conservation of this editing system across various species [21].

In addition to ADATs that use tRNA as a substrate for A-to-I and C-to-U deamination and ADARs that use double-stranded RNA as a substrate for A-to-I deamination, there are also CDARs (APOBECs) that function as mRNA-substrate C-to-U deaminases and CDATs that function as tRNA-substrate C-to-U deaminases, which belong to the deaminase superfamily [22]. APOBEC1, the first cloned cytidine deaminase, alters a Gln codon (CAA) to a stop codon (UAA) in *APOB* mRNA, producing a truncated protein called apoB48 instead of the full-length protein apoB100 [23]. Furthermore, CDAT8 facilitates the conversion of C8 to U8 of tRNAs in *Methanopyrus kandleri* to maintain the tertiary structure of tRNAs ( Figure 1) [ 24‒ 26].

In summary, the results of the deaminase phylogenetic analysis suggested that all the deaminases evolved from a bacterial ancestor with a typical deubiquitinating JAB-like domain that is capable of binding with metal ions and a nucleotide or similar molecule. These deaminases all belong to the Helix-4 deaminase superfamily [ 20, 27]. ADAT1 belongs to the evolutionary clade containing ADARs, whereas ADAT2 and ADAT3 belong to the APOBEC clade [21]. Further duplication of the ADAT1 gene after the emergence of metazoans may have produced ADAR and acquired dsRBDs, which are absent in ADAT1. Ultimately, the ancestral enzyme of eukaryotic ADAT2, ADAT3, and APOBEC is bacterial TadA [ 20, 21] It therefore appears that these enzymes are evolutionarily related through common ancestry but have diverged over time to adapt to distinct RNA substrates and functions.

## tRNA Editing by ADATs

**Figure 3:**
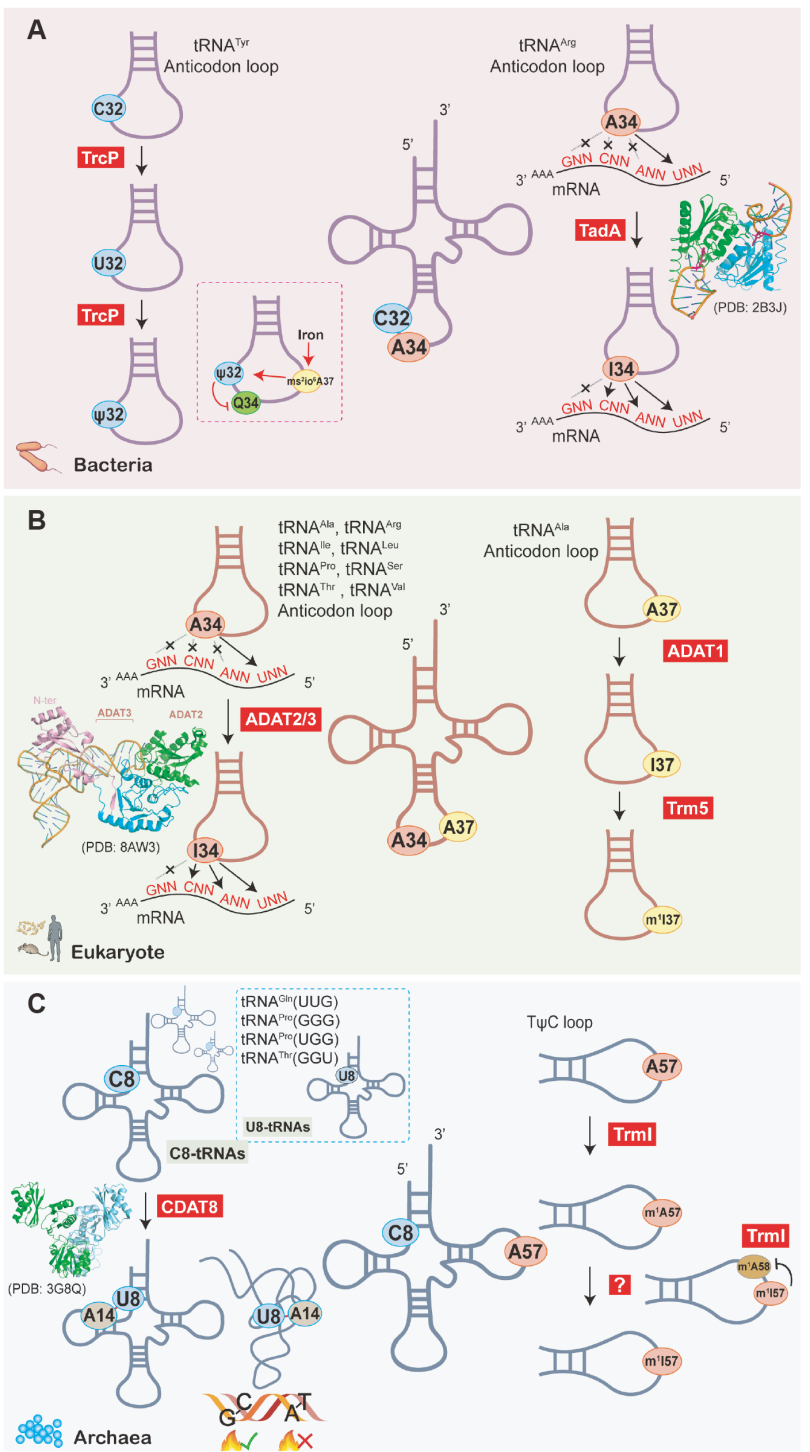
Figure 3 Editing of tRNA in different species (A) C32-to-U32-to-ψ32 modification by TrcP. ψ32 biogenesis is promoted by ms2io6A37, which is regulated by iron concentration. ψ32 partially suppresses Q34 formation. TadA-mediated editing of A34 to I34 extends the bacterial tRNAArg decoding capacity. The crystal structure of the Staphylococcus aureus TadA dimer with a tRNA anticodon loop (PDB: 2B3J) is shown. (B) A34-to-I34 editing by the ADAT2/3 complex to increase tRNA decoding capacity and A37-to-I37-to-m1I37 editing by ADAT1 and Trm5, respectively, in eukaryotes. The crystal structure of the Trypanosoma brucei ADAT2/3 heterodimer with tRNA (PDB: 8AW3) is shown [36]. (C) C8-to-U8 editing by CDAT8 to maintain normal tertiary structures while maintaining the genetic stability of M. kandleri in high-temperature environments and A57-to-m1A57-to-m1I57 modification by TrmI and an unknown enzyme in archaea. The crystal structure of the CDAT8 homodimer (PDB: 3G8Q) is shown.

RNA editing produces inosine on mRNA, which is recognized as guanine paired with cytosine, in both the translation machinery and the RNA splicing machinery [17]. Therefore, RNA editing leads to the generation of protein isoforms and the diversification of protein functions to improve the efficiency of the protein translation system. In addition to apoB100, which is produced by the premature termination of apoB48 due to RNA editing, as described above [23], which is a direct consequence of the translation step, the most typical example of the editing of the RNA splicing system is the editing of its own transcript by ADAR2 to create a new splice site. This leads to a shift in the open reading frame so that the stop codon is encountered prematurely, resulting in a truncated and destabilized protein, thereby reducing the functional ADAR2 [ 28, 29]. Similarly, tRNA editing in the anticodon has a direct impact on decoding. In the anticodon of bacterial and eukaryotic tRNAs, the editing reaction occurs at position 34. Consequently, tRNAs with A34 decode the U-ended codon only, whereas tRNAs with I34 decode the U-, A- and C-ended codons through wobble pairing ( Figure 3A,B) [ 30, 31], thus minimizing the number of necessary tRNA sequences encoded by the genome. Remarkably, the first case of four-way wobbling caused by adenosine and inosine was recently identified in *Bacillus subtilis*. The enzyme yaaJ, which is responsible for A-to-I deamination in tRNA ^Arg^(ICG), is not required for cell survival, but the absence of yaaJ induces frameshifting and affects growth and competence, which explains its conservation in *B*. *subtilis* [32].

### A-to-I editing in tRNAs

A-to-I deamination occurs at three positions of the tRNA, including positions 34 and 37 of the anticodon loop and position 57 of the T-loop. The I34 of eukaryotic tRNAs is catalyzed by ADAT2/3, which occurs in tRNA ^Ala^, tRNA ^Arg^, tRNA ^Ile^, tRNA ^Leu^, tRNA ^Pro^, tRNA ^Ser^, tRNA ^Thr^ and tRNA ^Val^. Conversely, the bacterial TadA dimer only catalyzes the deamination of A34 of tRNA ^Arg^ ( Figure 3A). TadA is the first prokaryotic RNA editing enzyme whose editing activity was reconstituted in 2002. TadA directly recognizes the anticodon stem-loop of tRNAs to catalyze the formation of I34 [13]. The cocrystal structure of *Staphylococcus aureus* TadA with RNA substrates shows that two TadA dimers bind with four RNA substrates to form an asymmetric unit. Each TadA monomer comprises five α-helices and five β-folds and contains an active center that binds with RNA and Zn ^2+^ [33]. The Zn ^2+^-bound water molecule in the active site is deprotonated by a catalytic glutamate residue, allowing hydroxide to attack the C6 position of adenosine. Concurrent protonation of N1 results in the formation of a high-energy intermediate, which is converted to inosine through a series of proton transfers and ammonia removal [ 34, 35]. Compared with the apo-structure of *Aquifex aeolicus* TadA without an RNA substrate, the changes in protein structure are modest [16]. However, the conformation of the tRNA anticodon loop (nucleotides 33‒37) undergoes conformational remodeling upon binding to TadA to avoid severe steric hindrance to the α5 helix of TadA [33]. In addition to bases 33-37, the unusual C32-A38 base pair that caps the loop forms extensive hydrogen bonds with TadA ( Figure 3A) [ 14, 33].

Yeast ADAT2 and ADAT3, which target tRNAs for the deamination of adenosine at position 34, were first described in 1999 [14]. ADAT2 contains a highly conserved Glu at position 56, which is an essential amino acid for proton shuttling during deamination, while ADAT3 has a Val residue at this position [14]. The TadA dimer and ADAT2/ADAT3 comparison showed that additional peptides are present at the C-terminus of ADAT2 and the N-terminus of ADAT3. In addition, the N-terminus of ADAT3 was found to contain a distinct RNA binding domain ( Figure 3B) comprising positively charged residues (Lys50, His70, Lys72 and Arg73) that are essential for tRNA binding [ 15, 36].

Resolution of the structure of the mouse ADAT2/3 complex has provided valuable information for proposing a mechanism for the deamidation of tRNA substrates by TadA and ADAT2/3. TadA directly recognizes the anticodon loop of tRNA to complete the deamination of tRNA ^Arg^. ADAT3 first binds to any tRNA after recognizing its 3-dimensional structure through its N-terminal domain. With the rotation of the N-terminal domain, the anticodon loops of tRNAs are then presented to the active site of ADAT2, which specifically recognizes the tRNA substrate to complete the deamidation reaction [37]. In *Saccharomyces cerevisiae*, the CGA codon is rare and inefficient [38]. Consecutive repeats of CGA codons have been reported to cause ribosome blockade of the mRNA, resulting in slower translation and reduced translation [ 39, 40]. The CGA codon is decoded by the A:I pairing of tRNA ^Arg^(ICG), which is formed by the deamination of tRNA ^Arg^(ACG) by yeast ADAT2/3, together with seven other tRNAs. However, the ratio of tRNA ^Arg^(ICG/ACG) resulted from ADAT2/3 activity in yeast is less than 30%, suggesting that the efficiency of decoding consecutive CGA codons may suffer from a tRNA ^Arg^(ICG) shortage [41].

Inosine at positions 37 and 57 is less common in organisms. I37 is found in eukaryotic tRNA ^Ala^ and is formed by deamination of the ADAT1 dimer, followed by methylation of Trm5 to form m ^1^I37 ( Figure 3B) [42]. In yeast, ADAT1 was discovered in 1996 [ 42, 43], showed remarkable similarities to ADARs and was able to catalyze adenosine deamination at position 37 of tRNA ^Ala^ [44]. In contrast, I57 is found only in archaea, where A57 is first modified to m ^1^A57 by TrmI before being deamidated by an unknown enzyme to form m ^1^I57 ( Figure 3C) [45]. The functions of I37 and I57 have not been well studied and remain unclear. At position 37, the presence of inosine suggests that it may regulate translation fidelity, while at position 57, it may be involved in stabilizing the tertiary structure of tRNA due to its location in the TψC loop. Furthermore, the deamidation of m ^1^A57 to m ^1^I57 could affect the efficiency of m ^1^A formation at position 58 ( Figure 3C) [46].

### C-to-U editing in tRNAs

C-to-U editing of tRNAs in eukaryotes is not widespread and has been identified only in organelles [47] and in the cytoplasm of *Trypanosoma brucei* [ 27, 48‒ 50 ] and *Arabidopsis thaliana* ( Figure 2) [51]. Extensive tRNA deamination of cytidine to uridine at position 8 was discovered in the hyperthermophilic archaeon *M*. *kandleri*. Typically, tRNAs retain a U at position 8 to form a Hoogsteen base pair with A14 to assist in the proper folding of L-shaped tRNAs [52]. In contrast, in *M*. *kandleri*, 90% of tRNAs have a C at position 8, except tRNA ^Gln^ (UUG), tRNA ^Pro^ (GGG), tRNA ^Pro^ (UGG) and tRNA ^Thr^ (GGU) which have a U8, which requires a C-to-U deamination reaction ( Figure 3C). The CDAT8 enzyme responsible for the deamination of C8 to U8 in *M*. *kandleri* was identified by the conserved His-Ala-Glu-X _n_-Pro-Cys-X-X-Cys motif [53] in the cytidine deaminase domain (CDD). CDAT8 consists of an N-terminal CDD, a central ferredoxin-like domain (FLD), and a C-terminal THUMP domain ( Figure 1) [54]. The CDD shares the core structure of well-established mRNA cytidine deaminases such as APOBEC2 [55]. Additionally, the FLD and THUMP domains display considerable homology with those of the prokaryotic ThiI enzyme or eukaryotic Tan1/THUMPD1, which is responsible for 4-thiouridine modification at position 8 (s ^4^U8) or acetylation at position 12 (ac ^4^C12) [ 56, 57]. CDAT8 forms an asymmetric homodimer that binds with the acceptor tRNA stem at the dimer interface. The FLD and THUMP domains of one monomer bind with the acceptor stem, thereby exposing C8 to the active site of the CDD of the other CDAT8 monomer, which specifically recognizes the 3′-CCA end and catalyzes the deamination reaction in the presence of Zn ^2+^ [25]. The reason that *M*. *kandleri* edits 30 out of 34 tRNA genes when CDAT8 is inserted into U8 is probably related to the need to stabilize its genome at the high temperatures to which it is exposed. Indeed, G:C pairing ensures better genome stability ( Figure 3C), as evidenced by the exclusive use of G:C base pairs in all of its tRNA stems [ 24, 50].

**Figure 4:**
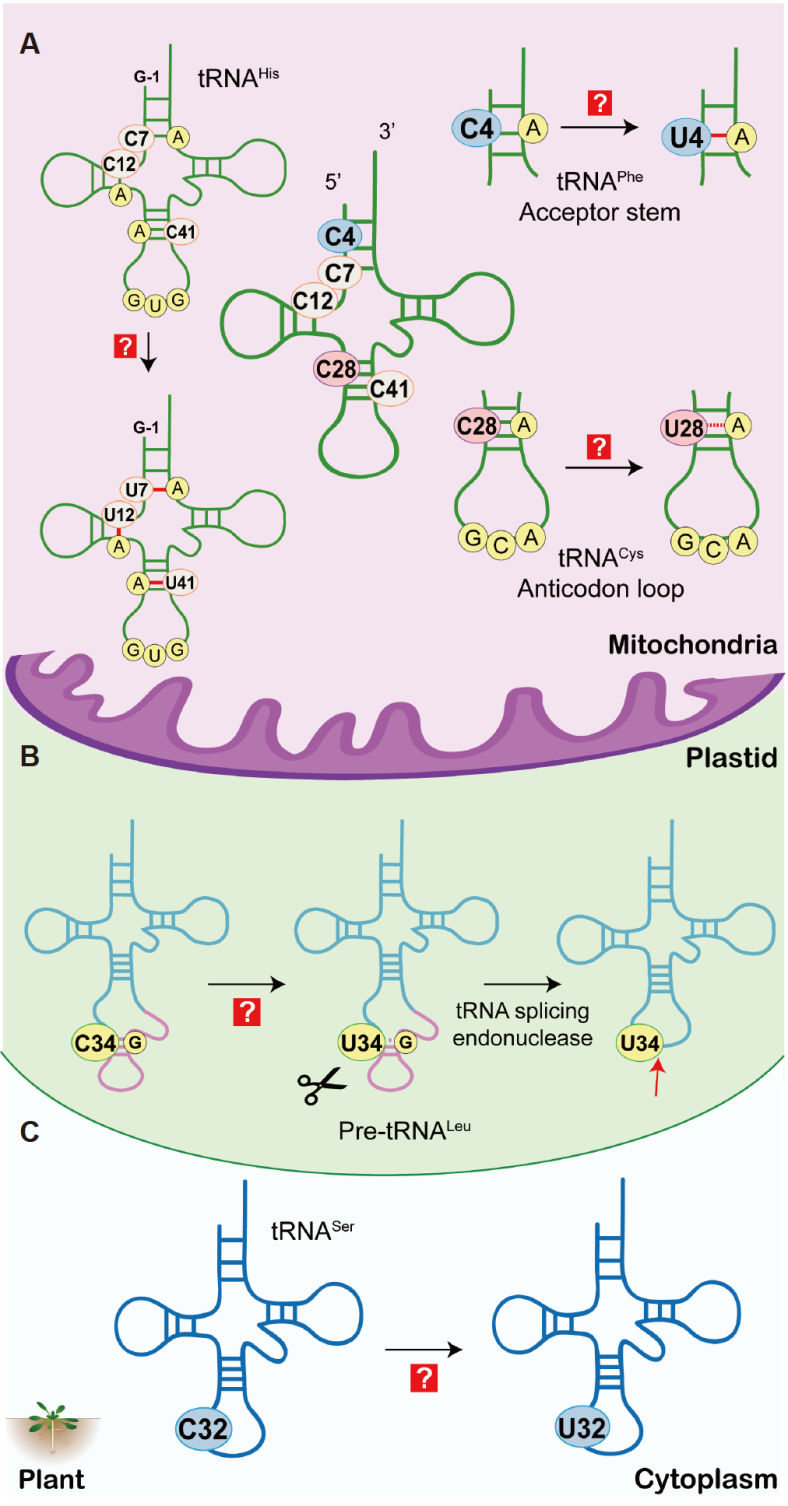
Figure 4 Editing of tRNAs in the mitochondria, plastids and cytoplasm of plants (A) C-to-U editing at positions 4, 7, 12, 28 and 41 of tRNAs by unknown enzymes in the mitochondria of plants. (B) C-to-U editing at the first position of the anticodon by an unknown enzyme to enhance intron splicing efficiency in the plastid. The intron is shown in pink. (C) C32-to-U32 editing by an unknown enzyme in the plant cytoplasm.

Plant mitochondria undergo extensive RNA editing, primarily of mRNAs [58], to provide the correct genetic information, with only a small amount occurring on tRNAs. In dicot and potato mitochondria, C-to-U editing occurs at position 4 of tRNA ^Phe^(GAA) to correct the C4:A64 mismatch in the acceptor stem [ 59, 60] and at position 28 of tRNA ^Cys^(GCA) to convert the C28:U42 mismatch in the anticodon stem to the non-canonical base pair U28:U42, which is a prerequisite for pseudouridylation ( Figure 4A) [ 59, 61]. In the mitochondrial tRNA ^His^(GUG) gene of the gymnosperm *Larix leptoeuropaea*, three C-to-U editing events are required to correct the C:A mismatches involving C7 in the acceptor stem, C12 in the D stem and C41 in the anticodon stem ( Figure 4A) [62]. In addition, for tRNA ^Phe^ and tRNA ^His^, editing occurs on precursor tRNAs before removing the 5′-leader and 3′-trailer sequences [ 60, 62, 63]. In the plastid of the moss *Takakia Lepidozioides*, the first base of the anticodon of pre-tRNA ^Leu^ (CAA) is edited from C34 to U34, forming the canonical tRNA ^Leu^(UAA). In this case, anticodon editing occurs before RNA splicing, showing that editing is a strict prerequisite for tRNA maturation ( Figure 4B) [64]. C32 in eukaryotic cytoplasmic tRNA ^Ser^ and tRNA ^Thr^ is usually methylated by Trm140/METTL2/METTL6/METTL8 family proteins [ 65‒ 68]; however, in *Arabidopsis thaliana*, cytosolic C32 of tRNA ^Ser^(GCU) and tRNA ^Ser^(AGA) undergo deamination to form U32 ( Figure 4C), which is otherwise not observed in fungi and higher eukaryotes. However, this reaction is not associated with any of the *At*ADAT1, *At*ADAT2, or *At*ADAT3 enzymes that are responsible for catalyzing the formation of I34 or I37 of tRNA ^Ala^, tRNA ^Leu^, *etc* . [51].

**Figure 5:**
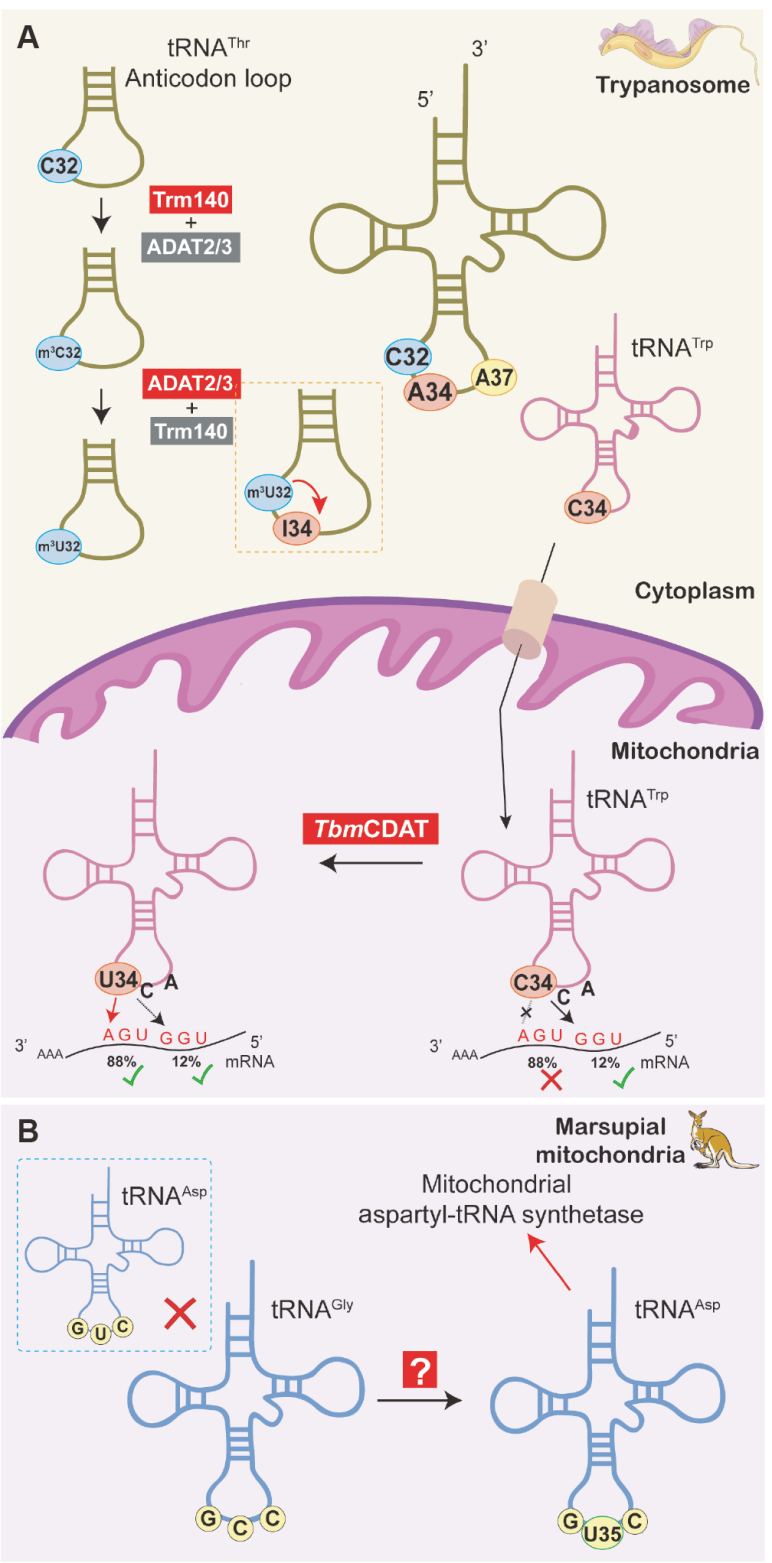
Figure 5 Special editing mode for trypanosomes and marsupial mitochondria (A) C32-to-m3 C32-m3U32 bound to Trm140 and the ADAT2/3 complex in the trypanosome cytoplasm could stimulate A34-to-I34 editing via the ADAT2/3 heterodimer (mentioned in Figure 3C). C34-to-U34 of tRNATrp is transported by TbmCDAT into the mitochondria to decode the UGA codon for tryptophan. (B) C35-to-U35 editing of tRNAGly to decode aspartic acid for recognition by mitochondrial aspartyl-tRNA synthetase in marsupial mitochondria. The red cross in the dashed box indicates that no mitochondrial tRNAAsp gene is encoded in the marsupial mitochondrial genome.

C-to-U editing at tRNA position 32 was first found to be catalyzed by ADAT2/3 in three tRNA ^Thr^ isoacceptors in the cytoplasm of *T*. *brucei* [ 48, 69 ]. This editing event requires methylation at position 32 by the methyltransferase Trm140, which ultimately results in the creation of m ^3^U32. During this process, Trm140 and ADAT2/3 interact continuously to carry out their respective enzymatic reactions ( Figure 5 A) [49]. Deamination at position 32 also stimulates A-to-I editing at position 34 and is considered to be essential for protein synthesis [ 48, 49]. There is also deamination of C34-to-U34 in the tRNA ^Trp^(CCA) of *T*. *brucei* mitochondria [70], which ultimately leads to the production of Um34 after 2′-O-methylation. Additionally, in the mitochondria of *T*. *brucei*, the reassigned UGA stop codon decodes tryptophan, whereas in the nucleus, there is only one tRNA ^Trp^(CCA) to decode the UGG codon [71]. Following mitochondrial import, approximately half of the nucleotides at position 34 of tRNA ^Trp^(CCA) undergo C-to-U editing to decode the UGA codon, and the balance of this ratio depends on the thiolation of U33 in this tRNA [72]. This deamination reaction is catalyzed by *Tbm*CDAT, which was identified as the responsible enzyme in 2021 ( Figure 5A) [27]. The marsupial mitochondrial tRNA ^Asp^ provides a similar illustration. Because marsupials lack tRNAs capable of decoding mitochondrial aspartic acid codons, C35 of tRNA ^Gly^(GCC) is deamidated to U35 so that it can be recognized by mitochondrial aspartyl-tRNA synthetase, ensuring proper translation ( Figure 5B) [73]. As with tRNA ^Trp^(CCA), approximately 50% of the mature tRNA ^Gly/Asp^ pool is found in an edited version of tRNA ^Asp^(GCC) that is specifically aminoacylated with Asp, while the unedited version remains aminoacylated with Gly [73].

The first case of editing cytidine to pseudouridine (C-to-ψ) at position 32 of tRNA ^Tyr^ in *Vibrio cholerae* has recently been documented. Unlike A37/57-to-m ^1^I37/57 or C32-to-m ^3^U32, which involve two separate enzymes that undergo methylation and deamination, the conversion of C-to-ψ is performed using a single enzyme, TrcP [74]. TrcP mediates C-to-U editing followed by U-to-Ψ conversion ( Figure 3A). Furthermore, the modification network in the tRNA ^Tyr^ anticodon loop influences the U-to-Ψ conversion. Indeed, C-to-Ψ editing is promoted by the methyl thiolation of A at position 37, similar to ms ^2^io ^6^A, which varies with environmental iron concentration and partially suppresses queuosine (Q) formation at position 34 ( Figure 3A). According to AlphaFold-2 [75], TrcP (ID: AF-Q9KN61-F1) consists of a cytidine deaminase, a pseudouridylase, and a long helical domain, where the unique long helical domain binds with the tRNA during both reactions ( Figure 1). Interestingly, TrcP does not possess the consensus motif of catalytic residues shared by deaminases but is structurally similar to zinc-dependent deaminases, suggesting that these deaminase catalytic pockets may have arisen via different evolutionary pathways [74].

## Other Functions and Clinical Significance of ADATs

Although tRNA is a common and widespread substrate for ADATs, it has been reported that the ADAT2/3 heterodimer of the protozoan *T*. *brucei* can deaminate C-to-U DNA [76]. As mentioned above, ADAT2/3 in *T*. *brucei* catalyzes the conversion of both C-to-U at position 32 and A-to-I at position 34 in tRNA ^Thr^(AGU), and the deamination of C at position 32 is stimulated by the formation of I34 [48]. Notably, trypanosomal ADAT2/3 is also involved in the C-to-U conversion of ssDNA but not in the A-to-I conversion, suggesting a possible link with the mechanism of antigenic variation that favors escape from the infected host immune system [76]. Interestingly, in the primitive chordate amphioxus *Branchiostoma japonicum*, the heterodimer ADAT2/3 ( *Bj*ADAT2/3) performs not only A-to-I editing of tRNA but also C-to-U and A-to-I deamination of DNA, providing a unique example of deaminase with tRNA and DNA editing capacity in metazoans [77]. *Bj*ADAT2/3 preferentially catalyzes A-to-I and C-to-U deamination in the loop of DNA hairpin structures. In addition, *Bj*ADAT2 has the potential to edit *E*. *coli* DNA and to target TCG and GAA sequences in the *E*. *coli* genome [77] .

**Figure 6:**
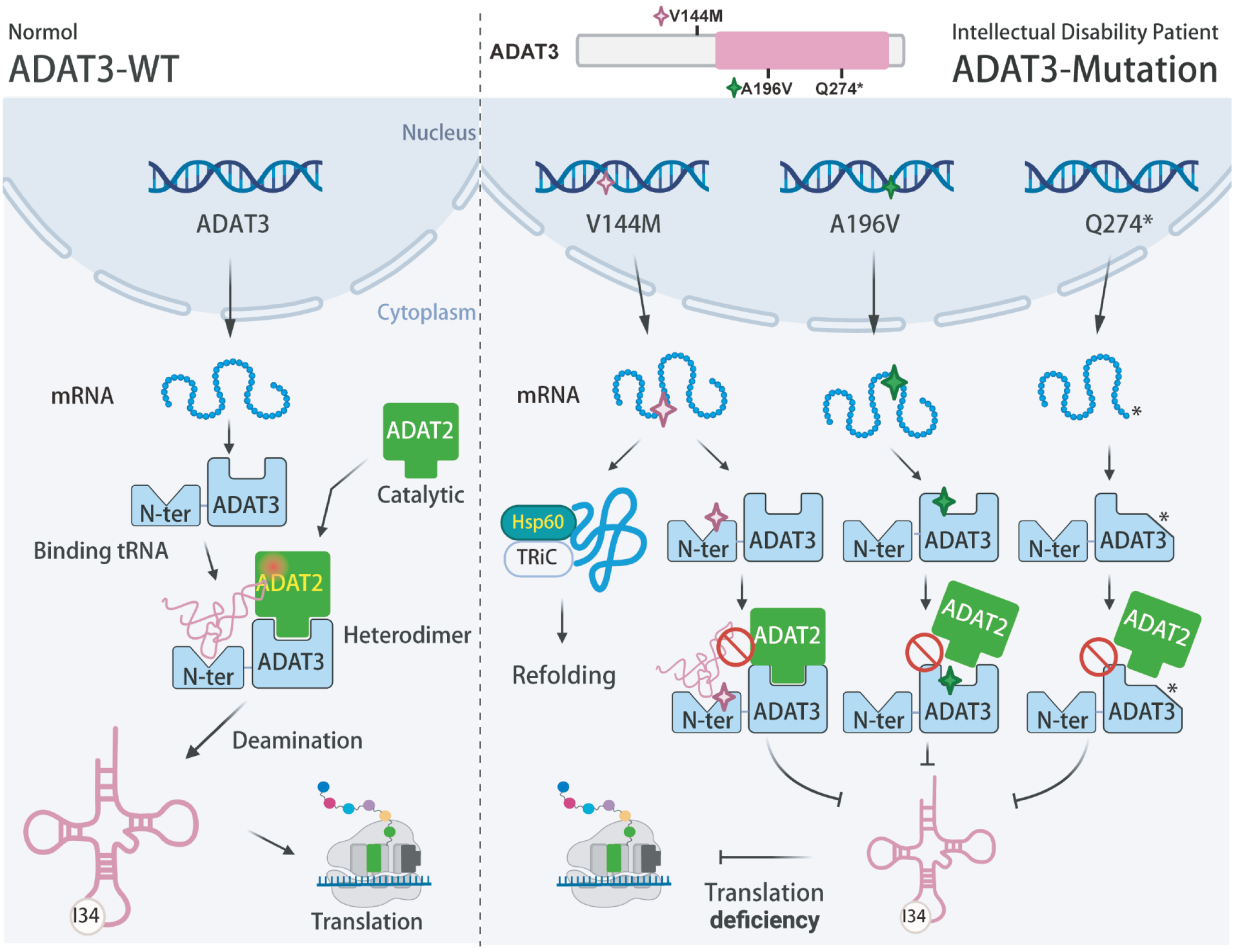
Figure 6 Schematic diagram of ADAT3-associated pathogenesis ADAT3 interacts with ADAT2 and functions as a cofactor for binding with tRNA substrates to enable ADAT2 deamination activity. ADAT2/3 dimers are localized in both the nucleus and the cytoplasm (not shown). ADAT3 variants have been identified in the cells of individuals with intellectual disability, in which the A196V (pink star) and Q274* mutants display impaired interactions with ADAT2, and the V144M mutant (green star) has a normal interaction with ADAT2 but is incapable of tRNA binding. All these defects lead to a reduction in the activity of the ADAT2/3 enzyme, ultimately leading to a reduction in I34-tRNA level. N-ter, N-terminal domain. The figure is created with BioRender.com.

In vertebrates, the deamination of DNA is catalyzed by activation-induced deaminase (AID), which belongs to a family of cytosine deaminases (APOBECs) [78]. All enzymes in this family act on cytosines in ssDNA or RNA and preferentially act on specific substrates. Combined with the preference of AID/APOBEC for cytidine in substrates, the ability of trypanosomes and amphioxus ADAT2/3 to catalyze the deamination of both adenosine and cytidine suggests an evolutionary function for this family of deaminases. An “APOBEC-mediated cascade model” has recently been proposed in which initial C-to-U events lead to a reduction in GC content and a reduction in the strength of neighboring RNA structures, triggering further C-to-U events. This is illustrated by the C-to-U deamination of SARS-CoV-2 viral RNA by APOBEC, which is at the origin of its accelerated evolution [ 79, 80]. Exome sequencing and autozygosity mapping have revealed several mutations in the human *ADAT3* gene that cause autosomal recessive intellectual disability (ID) [ 81‒ 83]. Recent studies have investigated the molecular and cellular effects of homozygous or biallelic heterozygous mutations (V144M, A196V and Q274*) in *ADAT3* in patients with intellectual disability who have a deficiency of edited tRNA (I34-tRNA) ( Figure 6). The valine residue at position 144 is conserved in *ADAT3* from yeasts to humans, and its mutation to Met was predicted to affect protein structure, resulting in aggregation. Although the steady-state level of ADAT3-V144M and its interaction with ADAT2 are unchanged, the tRNA-binding capacity of ADAT3-V144M is compromised. This leads to defects in the deaminase activity of the ADAT2/3 complex and ultimately to a reduction in inosine 34 in tRNAs. In addition, aberrant nuclear localization of the V144M variant was detected. Cells expressing ADAT3-V144M showed discrete cytoplasmic foci associated with targeting by heat shock protein 60 (HSP60) and TRiC/CCT chaperonin complexes [84]. On the other hand, mutation of the conserved residue 196 of ADAT3 from alanine to valine or premature translation termination (Q274*) in the deaminase domain similarly results in altered interactions with ADAT2 and consequent inability to maintain the I34-tRNA level, as well as the disruption of ADAT2-dependent nucleocytoplasmic distribution [85]. These studies show that a reduced level of inosine at tRNA position 34 can lead to intellectual disability in patients carrying pathogenic variants of ADAT3. Another observation showing the importance of the level of inosine modification is that human pluripotent embryonic stem cells and self-renewing cells optimize the translation of codons that depend on the inosine tRNA modification in the anticodon wobble position [86]. The importance of inosine in the wobble position of tRNA anticodon is thus clearly evident. Consistently, *ADAT2*/ *3* knockdown leads to cell death, and ADAT2/3 overexpression also affects cell survival. Although overexpression of ADAT2 has been shown to correct some of the defects in ADAT3 variant patients with intellectual disability, the level of inosine at the tRNA wobble position is tightly regulated *in vivo*, as mentioned above [41]. Therefore, increasing the amount of I34-tRNA by increasing the level of ADAT2/3 may not be an effective therapy for curing intellectual disability. Based upon these findings, there may be some potential therapeutic strategies, including the overexpression of ADAT3, to rescue inosine tRNA modifications. Functional studies should be performed to confirm the reversal of defects in inosine biogenesis caused by the overexpression of ADAT3 using cell and animal models. On the other hand, prenatal genetic screening targeting *ADAT3* may help to exclude embryos with pathogenic ADAT3 variants. Finally, it is conceivable that the gene editing of pathogenic mutations in *ADAT3* may be an intervention strategy for treating ADAT3-related diseases.

## Conclusion

RNA editing is an essential biological process for maintaining genetic information. It has been detected in various types of RNA, including mRNAs, tRNAs, and rRNAs, in mitochondrial and chloroplast-encoded RNAs, as well as in nuclear-encoded RNAs [13]. tRNA editing provides organisms with great flexibility at the translational level. Additionally, tRNA editing enzymes not only play a vital role in the maturation of tRNA but also determine the amino acid specificity of tRNA substrates in specific cases. Recently, it was discovered that some tRNA editing enzymes can recognize and edit DNA substrates, revealing the important evolution of tRNA editing enzymes as base editors for the correct expression of the genetic code. However, the editing enzymes responsible for the biogenesis of tRNA in many species remain unidentified. For instance, multiple C-to-U deaminases of tRNA in plant cytoplasm, mitochondria, and plastids, as well as the A-to-I deaminase at position 57 of tRNA in archaea, have not yet been identified ( Figure 2). It is essential to determine the molecular mechanisms and structural basis of ADATs across different species. Moreover, the biological functions of deamination at various tRNA sites remain unclear. Further exploration of tRNA editing enzymes, such as TrcP which mediates both C-to-U deamination and U-to-ψ of tRNA in *Vibrio cholerae*, is needed. Overall, the comprehensive study of tRNA editing and its related enzymes is an interesting and promising area of research.
